# Aphid parasitism and parasitoid diversity in cotton fields in Xinjiang, China

**DOI:** 10.1371/journal.pone.0207034

**Published:** 2018-11-08

**Authors:** Jinhua Li, Yuekun Wu, Qian Zhang, Haiqiang Li, Hongsheng Pan, Wei Lu, Dongmei Wang, Jianping Zhang, Yanhui Lu

**Affiliations:** 1 Xinjiang Production and Construction Corps Key Laboratory of Special Fruits and Vegetables Cultivation Physiology and Germplasm Resources Utilization/Key Laboratory at Universities of Xinjiang Uygur Autonomous Region for Oasis Agricultural Pest Management and Plant Protection Resource Utilization, Shihezi University, Shihezi, China; 2 State Key Laboratory for Biology of Plant Diseases and Insect Pests, Institute of Plant Protection, Chinese Academy of Agricultural Sciences, Beijing, China; 3 Institute of Plant Protection, Xinjiang Academy of Agricultural Sciences, Urumqi, China; 4 Xinjiang Agricultural University, Urumqi, China; Northwest A&F University, CHINA

## Abstract

Aphids are major pests of cotton crops in the Xinjiang Uygur Autonomous Region in China, and parasitoids are considered as important natural enemies in regulating aphid populations. However, information on aphid parasitoids in the Xinjiang cotton fields is limited, which hinders the study of aphid-parasitoid interactions and the application of conservation biological control against cotton aphids. In this study, a 3-year survey was conducted in a large geographical range that included three primary cotton planting areas in southern and northern Xinjiang. The population dynamics and the parasitism levels of an assemblage of aphids in the cotton fields were investigated along with the composition of the parasitoid community associated with these aphids. Aphid parasitization varied significantly within both years and seasons, with parasitism levels ranging from 0 to 26%, indicating that there is less effective biological control of parasitoids on aphids under field conditions. Among the primary parasitoids described, *Binodoxys communis* (Gahan) constituted 95.19% of the parasitoid species, followed by *Praon barbatum* Mackauer (3.15%), *Trioxys asiaticus* Telenga (1.01%) and *Lysiphlebus fabarum* Marshall (0.65%). Significant differences were found in the composition of the primary parasitoid species between the cotton seedling period (June) and the flowering period (July-August), and two more primary aphid parasitoids were found in the seedling period. Twelve hyperparasitoid species belonging to six genera were found in our study, of which *Pachyneuron aphidis* (Bouché), *Syrphophagus* species and *Dendrocerus laticeps* (Hedicke) were the dominant species. The composition of the hyperparasitoid community also differed significantly between the seedling and the flowering periods. The description of this parasitoid community-associated assemblage of aphids in cotton fields will facilitate the study of aphid-parasitoid interactions and promote the development of effective cotton aphid management strategies in Xinjiang.

## Introduction

Cotton is one of the most economically important crops in China [1, 2], and the cotton industry is the mainstay of Xinjiang Uygur Autonomous Region, the main area of cotton production in northwestern China [1, 3]. However, cotton production is limited by the damage caused by aphids and other pests. During the last 40 years, cotton aphids (especially *Aphis gossypii* Glover) have spread widely in Xinjiang, resulting in an increase in infected areas and great losses of cotton. Originally, approximately 700 ha was infested with aphids in Turpan in 1985, which coupled with aphid infestations in eastern Xinjiang, caused 500 t of yield loss in cotton, which expanded to 37,200 million ha of aphid-infested cotton in the following year [4]. Thereafter, aphids gradually spread to all cotton growing areas in Xinjiang. For example, in northern Xinjiang, aphid-related losses of 30% were reported in 1994, and cotton production was only 56 kg per ha. In Aksu, an area in southern Xinjiang, aphids infested 13,800 ha of cotton in 2000 [5]. In addition to cotton, *A*. *gossypii* and other aphids are crucial pests of citrus, peach and many important greenhouse-grown vegetables, leading to great economic losses in agriculture [6–8]. High mobility, high climatic adaptability, and high fecundity make aphids difficult to control.

Parasitoids are parasites that kill their insect hosts as a normal part of their development [9]. Parasitoids can regulate the populations of various aphids in different agricultural crop systems [10–12], and some parasitoids have been notably used as an option for biological control. The species *Aphidius gifuensis* Ashmead, for example, was successfully used to suppress *Myzus persicae* (Sulzer) in a tobacco field [13–15]. However, the application of parasitoids as the biological control agent is delicate, and the efficiency may interfere by other parasitoid species. Highly diverse parasitoids contribute to the complex interactions of the parasitoids communities associated with aphids. Parasitoid communities mainly consist of primary parasitoids and secondary parasitoids. Primary parasitoids lay their eggs directly into the aphid, and their progeny develop by feeding on aphid tissues, leading to the formation of a mummy [16]. Secondary parasitoids include the “true hyperparasitoids” such as some species of *Alloxysta* [17] that lay their eggs inside the body of the primary parasitoid larvae inside a live aphid and “mummy parasitoids”, such as *Dendrocerus carpenteri* (Curtis) [16, 18], that attack the primary parasitoid inside the mummified aphids [19]. In addition to the trophic interactions with aphids, parasitoids interact at both intra- and interspecific levels within parasitoid communities [19, 20, 21], and these interactions may affect the population dynamics of parasitoid species, alter their community structure and exert their impact on their aphid host population.

Accurate identification of different parasitoid species is the cornerstone of biological control and is also the foundation for exploring aphid-parasitoid interactions. The PCR-based molecular approaches have proven useful in the identification of parasitoids in aphid mummy and in the assessment of parasitism rates in nature [22, 23]. They are considered as the most promising technique in host-parasitoid association studies [22, 23]. Prior knowledge of parasitoid species could facilitate the design of parasitoid-specific markers, which allow us to detect these parasitoids in an accurate and rapid way [24–26]. Furthermore, knowledge of the composition and abundance of parasitoids provides information on the dominant species at each trophic level, which can be used to interpret aphid-parasitoid-hyperparasitoid interactions and become integrated into the management of aphids.

In Xinjiang, four aphids cooccur in cotton fields, including *A*. *gossypii*, *Acyrthosiphon gossypii* Mordvilko, *Aphis atrata* Zhang (recently corrected as *Aphis craccivora* Koch) and *M*. *persicae* [27–30]. However, the efficiency of parasitoids as regulators of these cotton aphids is poorly understood, and the application of pesticides has been the dominant method of aphid management [31–33]. Some preliminary information on cotton parasitoids in Xinjiang has been reported. Yang et al. [34] conducted an investigation in Manas, a county in northern Xinjiang, and he recorded ten species of Aphidiidae (including *Aphidius uzbekistanicus* Luzhetzk, *Aphidius smithi* Sharma et Rao., *Praon barbatum* Mackauer, *Trioxys asiaticus* Telenga, and *Lysiphlebus fabarum* Marshall), which were observed in various crops including cotton. In another survey conducted in southern Xinjiang, three parasitoids belonging to Aphidiidae were reared from aphid mummies [*Praon volure* (Haliday), *Ephedrus plagialor* (Nees), and *Ephedrus nacher* Quilis], all of which can attack *A*. *gossypii*, *M*. *persicae*, and some other aphids [35]. In these studies, while host aphids of these parasitoids were recorded, the levels of parasitism caused and the impact of parasitism on aphid densities were not. To date, there have been no systematic surveys of aphid parasitic wasps in the cotton fields in Xinjiang, China.

In this study, the population dynamics of an assemblage of aphids and their associated parasitism levels were monitored in cotton fields in northern and southern Xinjiang. Parasitoid species were recorded, and the composition of the parasitoid community on cotton aphids was determined to exploit the dominant parasitoids as part of a comprehensive strategy for aphid management.

## Materials and methods

### Ethics statement

No specific permits were required for the described field studies.

### Study area

The experiments were conducted across large geographical regions, including southern (Korla and Aksu) and northern (Changji) cotton planting areas. In each of these three areas, we have an experimental site (Korla: 41.75° N, 85.8° E; Aksu: 40.92° N, 80.37° E; Changji: 44.27° N, 87.32° E) for long-term monitoring arthropod population dynamics and community structure in crop fields of Xinjiang. In each area, three cotton fields were selected for this study. Individual fields, ranging from 1 to 2 hectares in size, were spaced at a minimum distance of 2 kilometers. The cotton was planted in early April, and agricultural practices common to the area were used, including growing protocols, drip irrigation, and plastic mulch, as described by Lu et al. [36]. No insecticides were used during the field trials.

### Aphid-parasitoid seasonal dynamics

The population of aphids and mummies was monitored from late May to early September in Aksu (2016 and 2017), Changji (2016 and 2017), and Korla (2015 to 2017). The surveys were conducted every seven days, and the surveyed area of each field was at least 10 m away from the border. On each surveyed date, five sites were selected randomly within a field, and at each site, the number of aphids and mummies were counted on 20 cotton plants. After the population survey, approximately 100 mummies were collected from five sites within each field.

### Parasitoid species composition

After field sampling, mummies were reared individually in 1.5 mL centrifuge tubes at 25 ± 1°C, 60% ± 5% RH (relative humidity), and a 16:8 h L:D (light: dark) photoperiod. Tubes were covered with nylon mesh for ventilation, and the mummies were checked daily for the emergence of adult parasitoids or hyperparasitoids. Emerged wasp specimens were examined individually and identified under a polarizing microscope (DM2500, Leica, Germany) using morphological characteristics [37–47]. Representative specimens were sent to experts for taxonomic confirmation. Mummies that did not produce wasps two months after the mummy collection were classified as intact mummies. All parasitoid species collected in this work were deposited at the Institute of Plant Protection, Chinese Academy of Agricultural Sciences, China.

### Statistical analysis

Populations of four aphids, including *A*. *gossypii*, *Ac*. *gossypii*, *M*. *persicae*, and *A*. *craccivora* were monitored in Aksu, Changji and Korla. Since these four aphid species coexist in cotton fields and because it was difficult to differentiate their mummies in the field, we recorded only the total number of these aphids (four species summed) for each sampling date. The parasitism rate, also referred to as the aphid mummification rate, was calculated by dividing the number of mummies (all parasitoids in all aphid species summed) found on the plants by the number of live aphids (all four species) plus the number of mummies. A mixed effect linear model (proc mixed) was used to analyze the variation in aphid density between different years and seasons (the seedling and flowering periods). The effect factors included year, season, and their interaction (year×season). The variation in aphid parasitism between years and seasons (the seedling and flowering periods) was also performed with a mixed effect linear model. Before the analysis, the data of aphid population were log_10_(x+1) transformed, and the percentages of parasitism were arcsin-square-root transformed. A chi-square test was performed to compare the proportions of the different parasitoid taxa, which included the four primary aphids and various hyperparasitoids collected in different growing seasons (the seedling and flowering periods). Analysis was performed in SAS 9.3 (SAS Institute Inc.Cary, USA).

## Results

### Aphid-parasitoid seasonal dynamics

The population abundance of the aphids fluctuated over time in cotton. The mixed effect linear model analysis showed that the population densities of aphids significantly varied in different years and seasons (the seedling and flowering periods), and the parasitism level also significantly varied in different years and seasons (Table 1).

**Table 1:** Statistics from the mixed effect linear model (MIXED) analysis of the population abundance and parasitism rate of cotton aphids in different seasons and years.

Parameters	Regions	Effects	N*df*	D*df*	*F*	*P*
Population abundance of aphids	Korla	Year	2	12	58.24	<0.0001
		Season	1	12	31.09	0.0001
		Year×Season	2	12	89.68	<0.0001
	Aksu	Year	1	10	16.86	0.0021
		Season	1	10	49.17	<0.0001
		Year×Season	1	10	0.12	0.7395
	Changji	Year	1	8	808.00	<0.0001
		Season	1	8	3970.76	<0.0001
		Year×Season	1	8	1743.18	<0.0001
Parasitism rate of aphids	Korla	Year	2	12	10.32	0.0025
		Season	1	12	163.84	<0.0001
		Year×Season	2	12	5.46	0.0206
	Aksu	Year	1	10	34.27	0.0002
		Season	1	10	82.11	<0.0001
		Year×Season	1	10	269.13	<0.0001
	Changji	Year	1	8	8.34	0.0203
		Season	1	8	816.30	<0.0001
		Year×Season	1	8	8.34	0.0203

Note: Different seasons included the seedling period (June) and the flowering period (July and August), and different years included 2015, 2016 and 2017. N*df* and D*df* were the numerator and denominator degrees of freedom, respectively. The significant level of *P* was α = 0.05.

**Fig 1:**
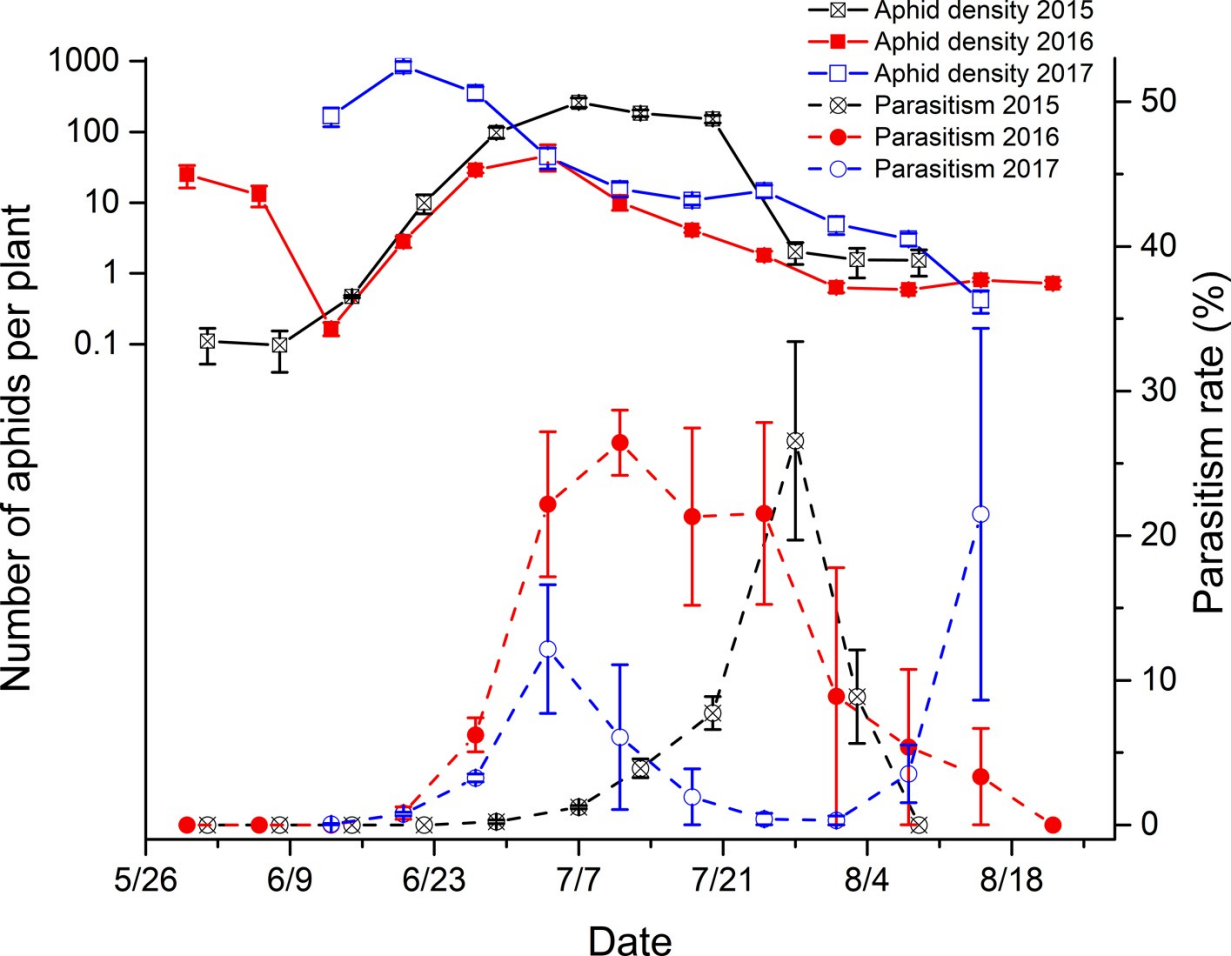
Population dynamics of cotton aphids and their level of parasitism in Korla, China (2015–2017). The data in the figures were shown as the mean ± SE.

In Korla, the fluctuation of the aphid population followed a somewhat similar pattern in 2015 and 2016, with the number increasing gradually and peaking in late June. The population in 2017 peaked at the earlier stage (middle June) and thereafter decreased gradually (Fig 1). The parasitoid populations were correlated with the aphid numbers but lagged behind the aphids. Specifically, in the initial stage, parasitism of the aphids increased continuously following the increase of the aphid population, reached the peak parasitism level 1 to 2 weeks after the peak aphid periods, and then decreased with the decrease of the aphid population. The parasitism levels in the seedling period (June) and the flowering period (July-Aug) were significantly different (2015: *df* = 12, *F* = -8.86, *P*<0.0001; 2016: *df* = 12, *F* = -8.61, *P*<0.0001; and 2017: *df* = 12, *F* = -4.7, *P* = 0.0005). The average parasitism rate of aphids in the flowering period was higher than that in the seedling period (2015: 8.05% ±1.67% in the flowering period and 0.04%±0.03% in the seedling period; 2016: 13.63% ± 2.13% in the flowering period and 1.41% ± 0.32% in the seedling period; and 2017: 6.56% ±1.00% in the flowering period and 1.35% ± 0.11% in the seedling period).

**Fig 2:**
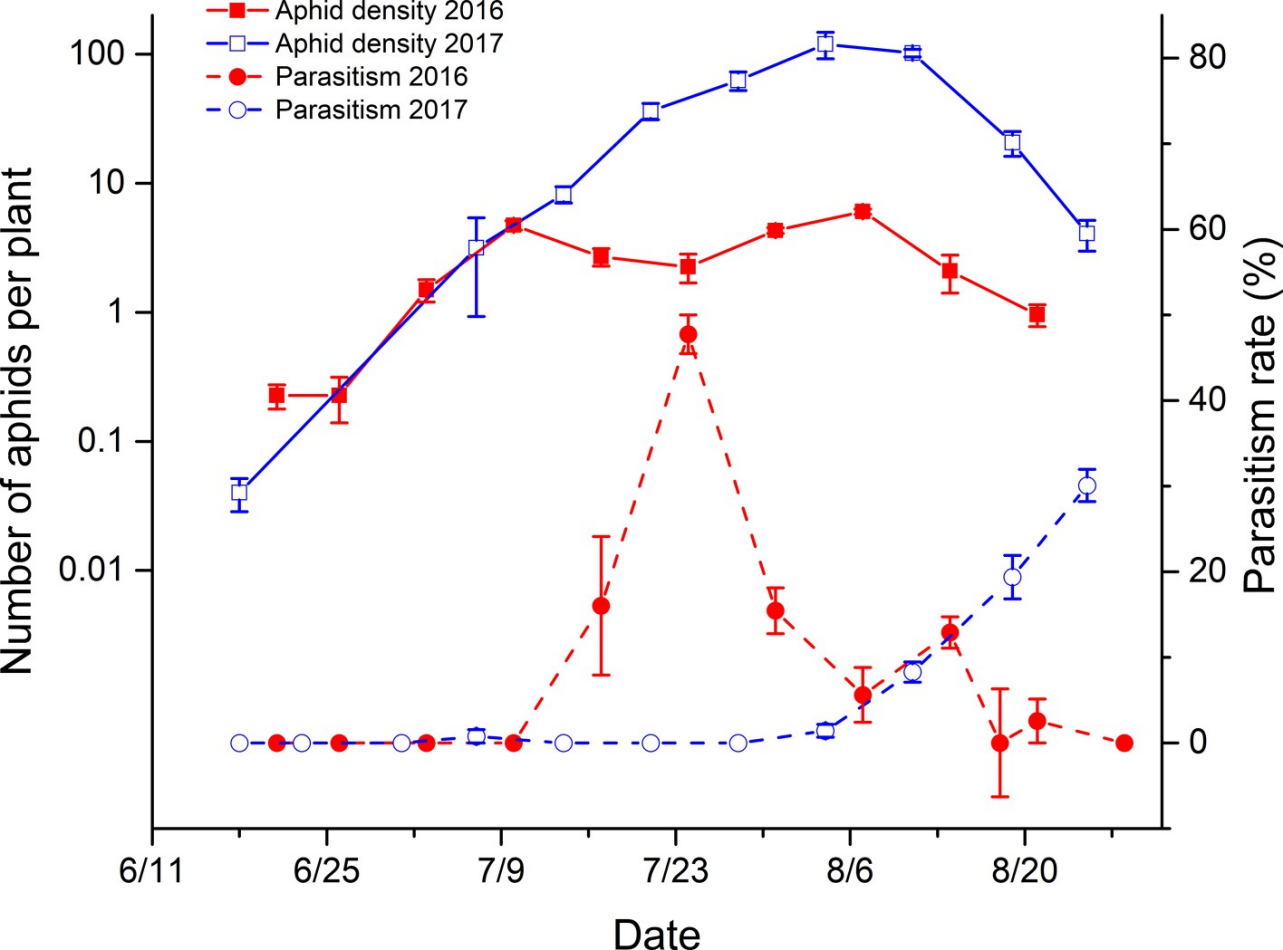
Population dynamics of cotton aphids and their level of parasitism in Changji, China (2016 and 2017). The data in the figures were shown as the mean ± SE.

In Changji, the aphid population increased and peaked in early July, decreased slightly, and elevated again, with a small peak in early August 2016. In 2017, after a continuous increase, the population reached its peak in early August and then decreased slowly. The parasitism rate increased over time as the aphid population increased but generally lagged (Fig 2), and the average parasitism levels of aphids in the seedling and flowering periods were significantly different (2016: *df* = 8, *F* = -22.24, *P*<0.0001; and 2017: *df* = 8, *F* = -18.16, *P*<0.0001). No mummies were found in the seedling period in either 2016 or 2017, and parasitism levels in the flowering period of those two years were 11.14% ±1.24% and 7.49% ± 0.39%, respectively.

**Fig 3:**
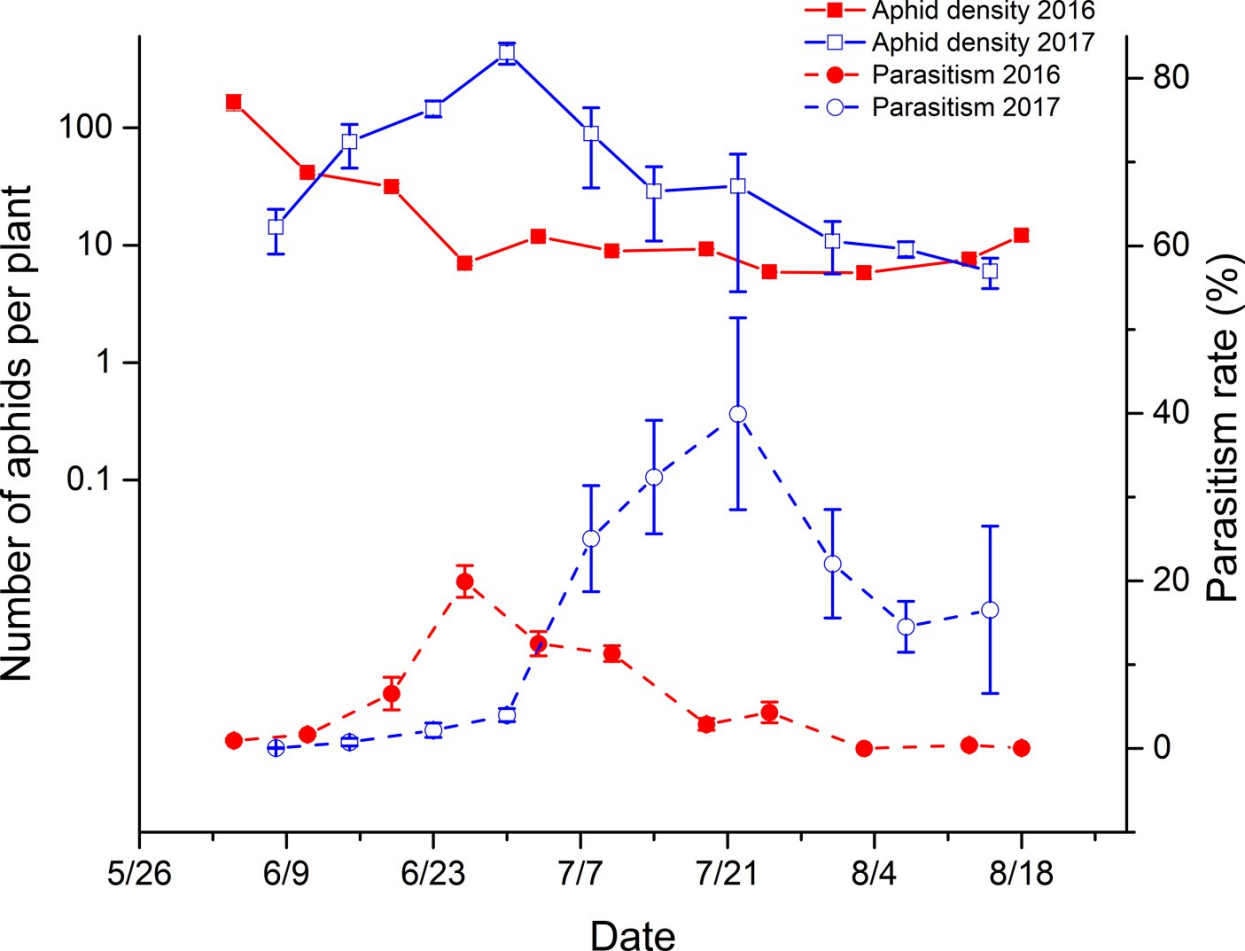
Population dynamics of cotton aphids and their level of parasitism in Aksu, China (2016 and 2017). The data in the figures were shown as the mean ± SE.

In Aksu, the aphid population peaked in early June and then reduced gradually in 2016. In 2017, it climbed to the peak in late June and then decreased slowly. The parasitism of aphids followed the fluctuation of the aphid population, and the peaks occurred in late June and late July in 2016 and 2017, respectively (Fig 3). The average parasitism rates of the two seasons (the seedling and flowering periods) were significantly different (2016: *df* = 10, *F* = 4.86, *P*<0.0007; and 2017: *df* = 10, *F* = -19.54, *P*<0.0001). The parasitism levels in 2016 were 8.31% ± 0.24% and 3.16% ± 0.15% in the seedling and flowering periods, respectively, and the levels in 2017 were 1.73% ± 0.40% in the seedling period and 25.08% ±1.68% in the flowering period.

### Parasitoid species composition

**Fig 4:**
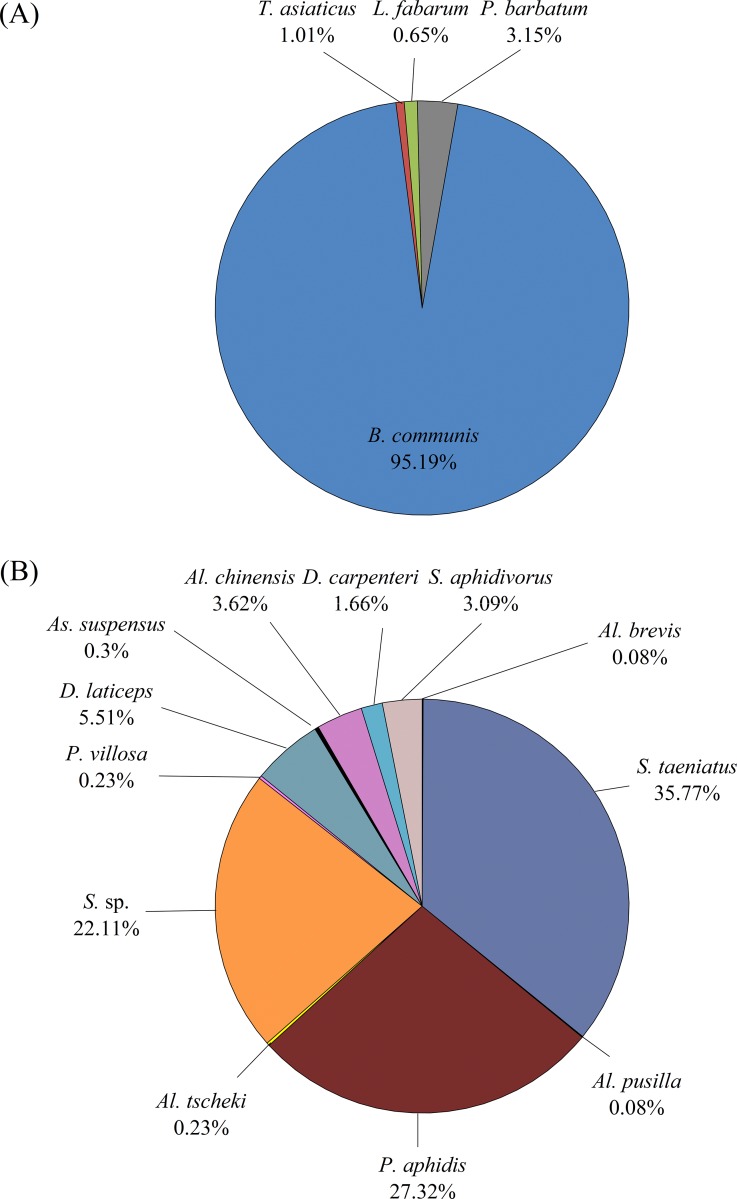
The composition of the primary parasitoids (A) and hyperparasitoids (B) associated with the cotton aphids in Xinjiang, China, in 2015–2017. Note: short name for the different primary parasitoids and hyperparasitoid species. Primary parasitoids: *B*. *communis*: *Binodoxys communis*; *L*. *fabarum*: *Lysiphlebus fabarum*; *T*. *asiaticus*: *Trioxys asiaticus*; *P*. *barbatum*: *Praon barbatum*. Hyperparasitoids: *Al*. *brevis*: *Alloxysta brevis*; *Al*. *chinsensis*: *Alloxysta chinensis*; *Al*. *tscheki*: *Alloxysta tscheki*; *D*. *carpenter*: *Dendrocerus carpenter*; *D*. *laticeps*: *Dendrocerus laticeps*; *P*. *aphidis*: *Pachyneuron aphidis*; *S*. *aphidivorus*: Syrphophagus aphidivorus; *S*. *sp*.: *Syrphophagus sp*.; *S*. *taeniatus*: *Syrphophagus taeniatus; P*. *villosa*: *Phaenoglyphis villosa*; *As*. *suspensus*: *Asaphes suspensus*; *Al*. *pusilla*: *Alloxysta pusilla*.

In the three years of this study, 3,799 parasitoids were collected from cotton fields in Xinjiang, consisting of four primary parasitoid species (65%), and twelve hyperparasitoids species (35%). Of the primary parasitoids, *Binodoxys communis* (Gahan) accounted for 95.19% of the total, followed by *P*. *barbatum* (3.15%), *T*. *asiaticus* (1.01%), and *L*. *fabarum* (0.65%). The twelve hyperparasitoid species were dominated by *Syrphophagus taeniatus* (Förster), *Syrphophagus* sp., *Pachyneuron aphidis* (Bouché), *Dendrocerus laticeps* (Hedicke), *Syrphophagus aphidivorus* (Mayr), and *Alloxysta chinensis* Fülöp & Mikó (Fig 4).

The overall composition of the primary parasitoid species in the three years differed significantly between the seedling period (June) and the flowering period (July and August) (χ^2^ = 94.72, df = 3, P <0.001). The species *B*. *communis* accounted for 96.30% and 93.91% in the two periods, respectively. Two more species, *L*. *fabarum* (1.21%) and *T*. *asiaticus* (1.89%) were more common during the seedling period than during the flowering period.

**Fig 5:**
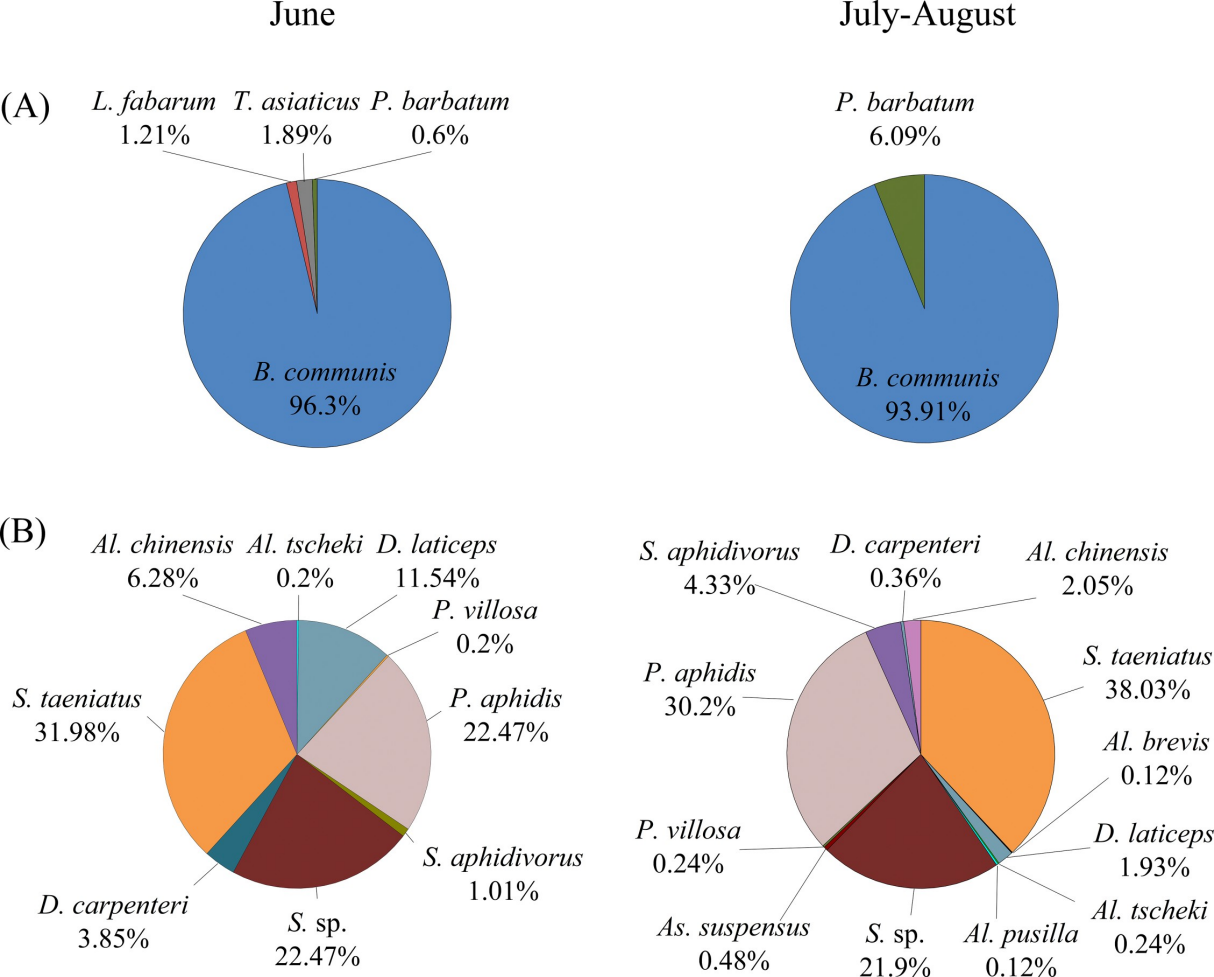
The cotton aphid primary parasitoid (A) and hyperparasitoid (B) diversity and composition in the seedling period (June) and the flowering period (July-August). Note: short name for the different primary parasitoid and hyperparasitoid species. Primary parasitoids: *B*. *communis*: *Binodoxys communis*; *L*. *fabarum*: *Lysiphlebus fabarum*; *T*. *asiaticus*: *Trioxys asiaticus*; *P*. *barbatum*: *Praon barbatum*. Hyperparasitoids: *Al*. *brevis*: *Alloxysta brevis*; *Al*. *chinensis*: *Alloxysta chinensis*; *Al*. *tscheki*: *Alloxysta tscheki*; *D*. *carpenter*: *Dendrocerus carpenter*; *D*. *laticeps*: *Dendrocerus laticeps*; *P*. *aphidis*: *Pachyneuron aphidis*; *S*. *aphidivorus*: *Syrphophagus aphidivorus*; *S*. *sp*.: *Syrphophagus sp*.; *S*. *taeniatus*: *Syrphophagus taeniatus; P*. *villosa*: *Phaenoglyphis villosa*; *As*. *suspensus*: *Asaphes suspensus*; *Al*. *pusilla*: *Alloxysta pusilla*.

The overall composition of the hyperparasitoid assemblage also differed significantly between the seedling and flowering periods (χ^2^ = 114.57, df = 11, P <0.001). The same species, with different relative proportions, were present in both periods, for example, *S*. *taeniatus* (31.98% in the seedling period and 38.03% in the flowering period), *P*. *aphidis* (22.47% in the seedling period and 30.2% in the flowering period), *D*. *laticeps* (11.54% in the seedling period and 1.93% in the flowering period), *Al*. *chinensis* (6.28% in the seedling period and 2.05% in the flowering period). Some hyperparasitoid species, such as *Alloxysta brevis* (Thomson), *As*. *suspensus* and *Alloxysta pusilla* (Kieffer), which account for <10%, were observed in the flowering period only (Fig 5).

## Discussion

Aphids are attacked by a number of species of Aphidiidae, which can result in population suppression [14, 48–52]. To enhance the application of parasitoids in aphid management, the species composition and dynamics of parasitoids should be explored. In this study, we provide a description of the parasitoid community attacking an assemblage of aphids in cotton fields in Xinjiang Province, China. The parasitoid community, based on the rearing of field-collected mummies, consisted of four primary parasitoid and twelve hyperparasitoid species.

The most important primary parasitoid, *B*. *communis*, is known to attack various aphids in different crops across many countries and regions [53–56]. Yang et al. [57] reported that among wasps emerged from mummies in northern China, *B*. *communis* accounted for most (>93%) of the primary parasitoids of *A*. *gossypii*. In our study, *B*. *communis*, representing an overwhelmingly high proportion of the primary parasitoids (>95%), showed their great potential to be a biocontrol agent of cotton aphids in Xinjiang. *B*. *communis*, together with *L*. *fabarum*, *T*. *asiaticus* and *P*. *barbatum*, constituted the primary parasitoid community of a pool of four aphid species. These other three parasitoid species were recorded in previous studies in Xinjiang, which were conducted in the 1980s [34, 58], but no mention was made of *B*. *communis*. Furthermore, the species *Lysiphlebia japonica* (Ashmead) was reported in earlier work as a common parasitic natural enemy of aphids in Xinjiang [59] but was absent in our study. The changes of the parasitoid species present in the cotton fields may be associated with the altering species composition of their hosts (aphid species) over the years. In Xinjiang, *Xerophilaphis plothikovi* Nevsky and *Aphis medicageinis* Koch were the major species of cotton aphids from the 1950s-1970s, and *A*. *gossypii* caused the first outbreak in 1985 and then increased as a dominant aphid species in cotton fields [60].

The links between primary parasitoids and hyperparasitoids are an important component of aphid-primary-hyperparasitoid trophic interactions, which may affect the biological control efficiency of primary parasitoids [61]. In the present study, of the twelve hyperparasitoids reported in the cotton fields, *P*. *aphidis* and species of *Syrphophagus* were the dominant parasitoids proportionally, which is similar to the results found in northern China with *A*. *gossypii* [57]. Some other species of hyperparasitoids, such as *D*. *laticeps*, *As*. *suspensus* and *Al*. *brevis*, that are found in the cotton fields of Xinjiang, were also recorded in wheat fields, soybean fields, or some forest habitats [19, 56, 62, 63] where they may be associated with various polyphagous primary parasitoids [16, 64–66]. Furthermore, two species, *Al*. *chinensis* and *Alloxysta tscheki* (Giraud), were recorded for the first time in the cotton fields in China.

In the present study, we found that the aphid populations showed periodic fluctuations, and aphid density varied significantly within study years and seasons. Many factors may affect aphid populations, such as seasonal changes in the weather conditions [67]. Temperature and humidity were also two important factors that influenced the population of *A*. *gossypii* and *Ac*. *gossypii* [28], and a high temperature (over 30°C) was reported to prolong development, increase mortality of the immature stages of *A*. *gossypii*, and reduce their fecundity [68]. The parasitism levels of the cotton aphids were also monitored in our study, and the increasing parasitism rate was related to and lagged with the corresponding increases in the aphids. The parasitism levels of the aphids, detected in our study, varied significantly within seasons and years, with an average parasitism level from 0 to 26%. Two more primary parasitoid species was present in the seedling period (June) than in the flowering period (July and August), which may relate to the population dynamics of *Ac*. *gossypii* and *A*. *craccivora*. In Xinjiang, *Ac*. *gossypii* and *A*. *craccivora* always occur in the early stages of cotton growth [28, 69–70], and the primary species *L*. *fabarum* and *T*. *asiaticus*, have been found to attack *A*. *craccivora* and *Ac*. *gossypii*, respectively [34,58]. The preference of the primary parasitoids to aphid hosts associated with the population dynamics of the aphids may contribute to the difference in the primary species found in the two periods. However, a significantly lower parasitism rate was found in the seedling period (except 2016 in Aksu), showing the inability of these parasitoids to suppress aphids in that period. Some strategies, such as intercropping and mixed planting, have been proved to effectively conserve natural enemies and enhance their biocontrol services. A cotton-wheat intercropping pattern aiming at aphid reduction in the seedling period was traditionally used in northern China, and the migration of the natural enemies from wheat to cotton after the wheat harvest led to increased productivity and reduced damage to cotton [71–73]. Parasitoids that can parasitize both cotton and wheat aphids, such as *A*. *gifuensis* in northern China, are of great potential benefit in cotton-wheat intercropping systems. Furthermore, the presence of flowering vegetation may lead to higher parasitism rates [74,75]. Thus, intercropping, mixed planting or other strategies may be an alternative to increase the parasitism levels of cotton aphids and is a topic that should receive further study.

An emerging molecular technique used in the study of host-parasitoid relationships allows us to track the aphid-parasitoid-hyperparasitoid trophic interaction in a highly efficient and comprehensive way [76, 77], and can reveal some interactions that were easily overlooked in traditional approaches, such as multiparasitism. Knowledge of the composition and abundance of parasitoids, which was achieved in this study can aid in the future selection of molecular markers for the identification of these parasitoids, and may help us interpret aphid-parasitoid-hyperparasitoid interactions and enhance the biological control of aphids.
